# Supplementary material for: Lipid Droplet Accumulation Independently Predicts Poor Clinical Prognosis in High-Grade Serous Ovarian Carcinoma
Source: Cancers (Basel). 2021 Oct 19;13(20):5251. doi: 10.3390/cancers13205251 (PMC8533764; doi:10.3390/cancers13205251)
Supplement: Supplementary file 1 [file cancers-13-05251-s001.zip › cancers-1425650-supplementary.pdf]

Supplemental Materials

Lipid droplet accumulation independently predicts poor clinical prognosis in high-grade serous ovarian carcinoma

Naoyuki Iwahashi,[1,6] Midori Ikezaki,[2,6] Masakazu Fujimoto,[3] Yoshihiro Komohara,[4] Yukio Fujiwara,[4] Madoka Yamamoto,[1] Mika Mizoguchi,[1] Kentaro Matsubara,[2] Yudai Watanabe,[2] Ibu Matsuzaki,[5] Shin-ichi Murata,[5] Yoshito Ihara,[2] Kazuhiko Ino,[1] and Kazuchika Nishitsuji[2,*]

[1]Department of Obstetrics and Gynecology, Wakayama Medical University, 641-8509 Wakayama, Ja-pan; naoyuki@wakayama-med.ac.jp (N.I.); madoka-y@wakayama-med.ac.jp (M.Y.); ma-ika@wakayama-med.ac.jp (M.M.); kazuino@wakayama-med.ac.jp (K.I.); [2]Department of Biochemistry, Wakayama Medical University, 641-8509 Wakayama, Japan; ikeza-ki@wakayama-med.ac.jp (M.I.); d1766082@wakayama-med.ac.jp (K.M.); d1766099@wakayama-med.ac.jp (Y.W.); y-ihara@wakayama-med.ac.jp (Y.I.), nishit@wakayama-med.ac.jp (K.N.); [3]Department of Diagnostic Pathology, Kyoto University, 606-8507 Kyoto, Japan; fuji-masa@kuhp.kyoto-u.ac.jp (M.F.); [4]Department of Cell Pathology, Graduate School of Medical Sciences, Kumamoto University, 860-8556 Kumamoto, Japan; ycomo@kumamoto-u.ac.jp (Y.K.); fuji-y@kumamoto-u.ac.jp (Y.F.); [5]Department of Human Pathology, Wakayama Medical University, 641-8510 Wakayama, Japan; m_ibu@wakayama-med.ac.jp (I.M.); smurata@wakayama-med.ac.jp (S.-i.M.);
[6]These authors contributed equally to this work.
*Correspondence: Kazuchika Nishitsuji, PhD, Department of Biochemistry, Wakayama Medical Univer-sity, 811-1 Kimiidera, Wakayama 641-8509, Japan, E-mail: nishit@wakayama-med.ac.jp; Phone/fax: +81-73-441-0628

Supplemental Procedures

Analysis of proliferation, lipid droplet (LD) accumulation, and adipophilin (ADP) expression

OVCAR-3 cells were cultured with different concentrations of glucose (2.25, 4.5, or 9.0 g/l) or in the absence or presence of K604 (10 μM), TMP-153 (200 nM; Cayman Chemical, Ann Arbor, MI, USA), Sandoz 58-035 (5 μM; Santa Cruz Biotechnology, Dallas, TX, USA) , or amidepsine A (20 μM; Cayman Chemical) for 72 h 37 °C, after which cell viability was determined by using the WST-1 cell proliferation assay (Roche, Basel, Switzerland) according to the manufacturer's protocol.

LDs were visualized by using the fluorescent probe Lipi-Green according to the manufacturer's procedure (Dojindo, Kumamoto, Japan). ADP expression was analyzed by means of Western blotting and immunocytochemistry with an anti-ADP antibody (LifeSpan Biosciences, Seattle, WA, USA). For Western blotting, OVCAR-3 cells were plated on 6-well culture plates and cultured as described above. Whole-cell lysates were prepared by means of the trichloroacetic acid precipitation method. Briefly, cells were washed three times with phosphate-buffered saline (PBS) and were then fixed with 10% (w/v) trichloroacetic acid in PBS on ice for 30 min, after which samples were centrifuged at 3000 × g for 15 min at 4 °C. Precipitates were dissolved in sodium dodecyl sulfate-polyacrylamide gel electrophoresis sample buffer [0.125 M Tris-HCl; 4% (w/v) SDS, 20% (v/v) glycerol, 12% (v/v) 2-mercaptoethanol, and 0.01% (w/v) bromo-phenol blue]. Lysates were subjected to sodium dodecyl sulfate-polyacrylamide gel electrophoresis with 5–20% gradient gels and were then transferred to polyvinylidene difluoride membranes (Millipore, Billerica, MA, USA). ADP on the membranes was probed with an anti-ADP antibody (1:1000) followed by use of a horseradish peroxidase-labeled anti-rabbit antibody (1:10,000; Cell Signaling Technology) and ImmunoStar LD (Wako Pure Chemical). Protein contents of cell lysates were normalized to β-actin expression levels. Signals were detected by using a WSE-6100 LuminoGraph I (ATTO Corporation, Tokyo, Japan). For immunocytochemical analysis, OVCAR-3 cells were plated on poly-l-lysine-coated cover glasses and cultured as described above. Cells were then fixed with 4% paraformaldehyde in PBS for 20 min at room temperature. After being washed three times with PBS, cells were blocked and permeabilized with Animal-Free Blocker (Vector Laboratories, Burlingame, CA, USA) containing 0.05% saponin for 20 min at room temperature. They were then incubated with an anti-ADP antibody (1:100) followed by a Cy3-conjugated secondary antibody (1:1000; Jackson Immunoresearch, West Grove, PA, USA). Specimens were mounted with Vibrance Antifade Mounting Medium with DAPI (Vector Laboratories) and were examined with an LSM700 microscope (Zeiss,

Oberkochen, Germany).

Analysis of LD accumulation and ADP expression in OVKATE cells

Another HGSOC cells, OVKATE cells were from the Japanese Collection of Research Bioresources Cell Bank (Osaka, Japan) and maintained in RPMI 1640 (Wako Pure Chemical, Osaka, Japan) supplemented with 10% heat-inactivated fetal bovine se-rum (Biowest, Nuaillé, France), 100 U/ml penicillin, and 100 μg/ml streptomycin (Wako Pure Chemical) at 37 °C in a 5% CO_2 atmosphere. OVKATE cells were cultured with different concentrations of glucose (0, 2.25, or 4.5 g/l), and LD accumulation and ADP expression were analyzed as described above.

Statistical analysis

Statistical comparisons were performed by means of the unpaired Student's t test or one-way analysis of variance followed by Dunnett's test or Bonferroni's test by using Prism software (GraphPad Software, Version 7.04, La Jolla, CA, USA). Results were said to be significant when p values were less than 0.05.

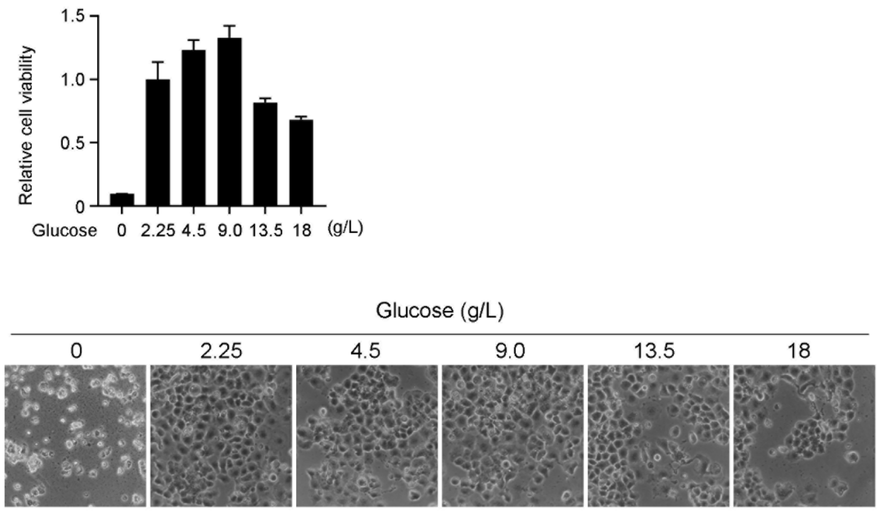

Supplemental Figure S1 Effect of glucose concentrations on proliferation of OVCAR-3 cells

OVCAR-3 cells were cultured at various glucose concentrations for 72 h, and proliferation was analyzed by using the WST assay. Phase contrast images of OVCAR-3 cells at each glucose concentration are also shown.

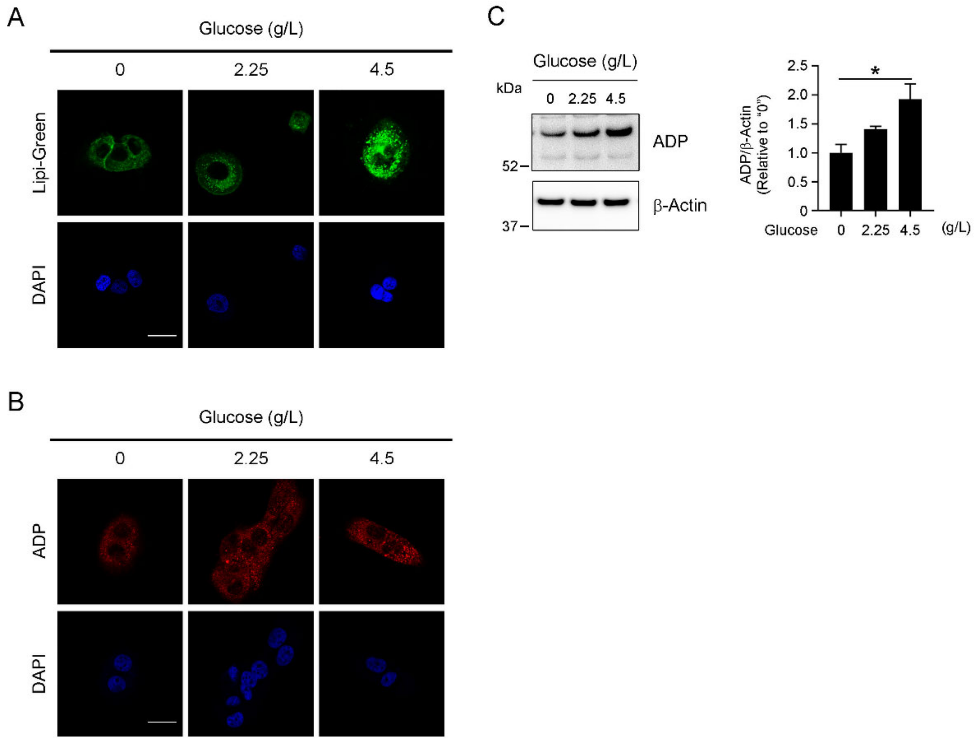

Supplemental Figure S2 Glucose-dependent LD formation in OVKATE cells. A OVKATE cells were cultured with 0, 2.25 g/l, or 4.5 g/l g/l glucose for 72 h, after which LDs in OVKATE cells were visualized by using the lipophilic Lipi-Green. **B** After being cultured as described in (A), cells were fixed with 4% paraformaldehyde and immunostained with an anti-ADP antibody. Scale bars: 20 μm. **C** After being cultured as described in (A), ADP expression was analyzed by means of Western blotting with the anti-ADP antibody. Data are means ± S.E. of three independent experiments. β-Actin was used as a loading control. *$P < 0.05$.

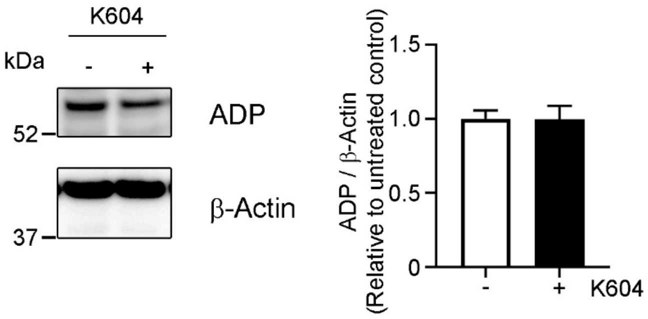

Supplemental Figure S3 No effect of K604 (10 μM) treatment on ADP protein levels. OVCAR-3 cells were treated with K604 (10 μM) for 72 h, after which ADP expression was analyzed by means of Western blotting. Data are means ± S.E. of three independent experiments. β-Actin was used as a loading control.

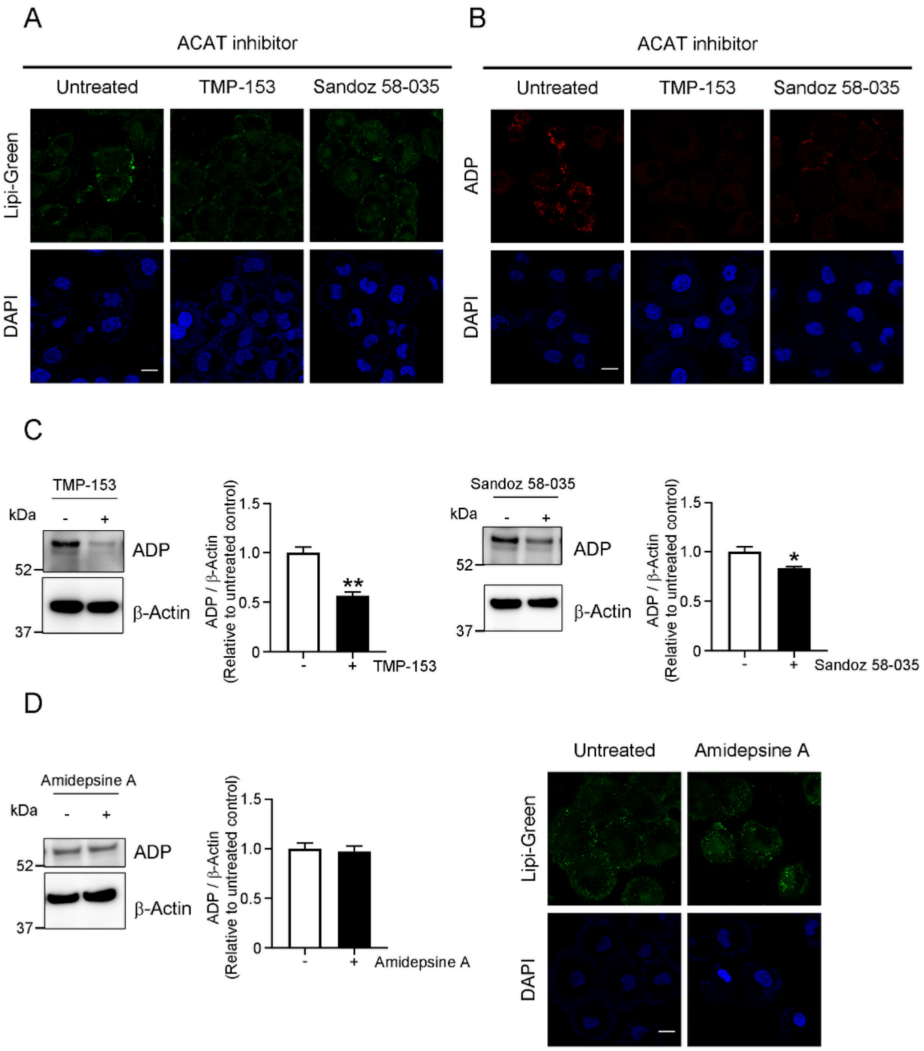

Supplemental Figure S4 Effects of TMP-153 and Sandoz 58-035, acyl-coenzyme A: cholesterol acyltransferase (ACAT) inhibitors, and amidepsine A, an acyl CoA: diacylglycerol acyltransferase inhibitor, on LD formation and ADP protein levels in OVCAR-3 cells.

OVCAR-3 cells were treated with TMP-153 (200 nM) or Sandoz 58-035 (5 μM) for 72 h, after which LD formation and ADP expression were analyzed by using Lipi-Green (A), ADP immunostaining (B), and Western blotting with an anti-ADP antibody (C). (D) OVCAR-3 cells were treated with amidepsine A (20 μM) for 72 h, after which LD formation and ADP expression were analyzed by using Lipi-Green and Western blotting, respectively. Scale bars: 20 μm. Data are means ± S.E. of three independent experiments. β-Actin was used as a loading control. *$P < 0.05$; **$P < 0.01$.
